# Hydrocyanic Acid in the Treatment of Insanity

**Published:** 1864-02

**Authors:** Kenneth McLeod


ART. II.—*Hydrocyanic Acid in the Treatment of Insanity.* By KENNETH McLEOD, M. D.

Among many trials of the efficacy of particular medicinal agents and modes of remedial treatment in particular forms of mental derangement, which I have instituted since coming into asylum practice, I have made a careful series of experiments into the effect of hydrocyanic acid in allaying cerebral irritation and excitement. The results which I have obtained are, upon the whole, so saisfactory, that although I should desiderate a more extended and minute induction for the sake of indicating exactly the cases and circumstances in which it is most suitable, still I consider it better to give them publicity, in order that the use of the drug, if it is as valuable and effectual as I believe it to be, may be extended, and that its merits and defects may become a subject of inquiry at the hands of others. As far as I have been able to learn by reading and personally inquiring into the practice of other asylums, though prussic acid is, in over-dose, the cause of such immediate and striking effects upon the brain, its moderate use has not been applied to any form of disease of that organ whose activities it is capable of extinguishing in less than minute.

Aware of the fallacies which are so liable to complicate investigations into the modes of action of therapeutical agents, and to cripple, nullify, or falsify results, I have with care attempted to guard against them. The most common sources of fallacy are—

1. The natural course and resolution of the disease after removal of a cause or causes, or the completion of the changes which constitute its term.

2. The effects of regimen, diet, moral treatment, and other circumstances operating simultaneously with the administration of the remedy as causes in a course of events.

3. The effect of other remedial agents given at the same time or previously.

4. The tendency to anticipate results and to "explicate appearances—not as they are, but as the observer pleases."

I have endeavored to obviate these, which attach largely to a practice in insanity, by—1st. Estimating the probable issue from a consideration of existing circumstances and symptoms, and comparison with other similar cases. 2d. Making due allowance for the effect of other means in existence as causes of cure. 3d. Administering the drug void of combination. And, 4th. Cultivating a salutary scepticism as to results, and taking rather the unsolicited testimony of other observers than depending solely on my own observation.

The evidence which I possess of its action has been gained from—1. Personal observation, as constant and close as possible. 2. The observations of attendants upon the insane. 3. The statements of other patients; and, 4. The admission, in several cases, of the patients themselves.

The trials have extended over six months, and the number of patients who have for a longer or shorter period been under treatment with it exceeds forty. I shall, in what follows, consider in order—1. The circumstances and cases in which the drug has been administered. 2. The effects of the administration. 3. The preparation, dose, and mode of administration employed; and 4. The indications derived from my experience of it, of its further use or trial; and give, shortly, a few of the most remarkable illustrative cases.

I. The feature of symptom which has in every case indicated the administration of the drug as a reputed calmative, is excitement—the manifested excess of cerebral activity which almost invariably accompanies, or assists in constituting, most forms of acute insanity, however caused or conditioned.

This increase of manifested energy may consist in an excessive activity of any or all of the representative faculties, gesture, feature, voice, or an intensified action of the brain itself, resulting in a morbid rapidity of ideation.

A simple increase of the evolution of nerve force, causing a more rapid rate of brain action and greater intensity of representation in the form of muscular acts, when excited by sufficient motive, and devoted to any end or a rational end, is a phenomenon of sound psychological action, and is manifested as emotion, passion, etc.; but when it exists in excess, without an adequate motive or any motive at all, and is not, consequently, devoted to any rational end or any end at all, it constitutes a pathological fact of the same sort, as every other pathological action or phenomenon characterised by excessive activity in a particular direction. Beyond recognising this excessive and sakeless cerebral vigor, or hypernoia, as it may be appropriately termed, as a simple, ascertained pathological fact, we cannot go; and, admitting it as such, we instinctively look for its conditions and causes, and, in the way of treatment, strive either to remove the cause, or introduce new causes—the knowledge of the causes and conditions of the pathological manifestation, as well as the causes and conditions which will remove it, being matter for investigation.

The *hypernoia* may co-exist with more or less mental derangement. It may be an utter delirium, in which reason and design are totally wanting, or may exist along with incoherence and delusions of all sorts and degrees, and with one or several active propensities, erotic, destructive, dirty, malevolent, homicidal, suicidal, etc. It forms the element of acuteness in many different forms of insanity, is the main object of the exhibition of medicines and plans of remedial treatment, morphia, antimony, warm bath, douche, emetic, purge, etc. Its degree measures alike the gravity of the disease, and the success of treatment; its abatement is a token of amelioration, and removal a triumph; the treatment of the faculty disorganization or *paranoia* being subsequently accomplished mainly by tonic, dietetic, and moral means.

The particular forms of insanity in which I have employed this remedy are—

| | | Cases. |
|---|---|---|
| 1 | Mania, acute | 13 |
| 2. | " chronic | 2 |
| 3. | " chronic, acute paroxysms | 2 |
| 4. | " menstrual | 2 |
| 5. | " puerperal | 2 |
| 6. | " recurrent | 1 |
| 7. | " epileptic | 2 |
| 8. | " epileptic, with menstrual excitement | 2 |
| 9. | " with hemiplegia | 2 |
| 10. | " with general paralysis | 5 |
| 11. | " with chronic hydrocephalus | 1 |
| 12. | Melancholia, acute | 3 |
| 13. | " chronic, with acute paroxysms | 3 |
| | | 40 |

II. The *effect* in every case has been very manifest. It has been almost purely psychal, consisting in a very remarkable, sudden, or gradual cessation of hypernoetic manifestations, with or without the induction of sleep. While its repeated exhibition has never failed to have some calmative effect, this has varied, according to the circumstances of the case, and has occurred in all degrees from the gradual, slight, and temporary, to the immediate, absolute, and permanent.

1. In cases of mania and melancholia of great severity and long duration, with organic disease of the brain and body, its calmative action has been more slowly produced, with more difficulty maintained, more evanescent and futile.

2. In recent cases of mania and melancholia, where no grave structural change exists, and the morbid condition has not become so stereotyped by constant repetition of similar changes, its exhibition has been followed by an immediate and sustained change for the better.

3. In the violent, paroxysmal mania of epilepsy and general paresis, in menstrual mania, and acute melancholic paroxysms, a single administration, or a few full doses at short intervals, have effectually dispelled the paroxysm.

The effect is thus of two sorts: 1. Immediate. In a few minutes, one to five generally, a patient who has just been shouting, chattering, dancing, swearing, thumping, &c., &c., becomes settled and quiet, sits upon a seat, and perhaps falls into a sound sleep. And 2, gradual; the patient becoming, as the hypernoia is thus, from time to time, warded off, more rational, companionable, and useful. While changes in psychal manifestation are thus very obvious and striking, observed and appreciated by attendants, and confessed to by patients themselves, who, on being questioned, admit the calmative action, and conferred power of self-control, concomitant physical phenomena are very obscure or wanting. Only in two cases have I observed a very decided change in the character of the pulse, which became slower, weaker, and, in one, slightly irregular; but this is probably owing to the difficulty of accurately observing it in such circumstances. In two other cases in which a slight over-dose was given, a semi-comatose condition was induced, with complete adynamia, partial ptosis, the accumulation of frothy saliva, pallor, slight affection of breathing and pulse, phenomena almost exactly resembling those immediately preceding an epileptic paroxysm. In a few cases the subjective sensations were described as—1. Slight transient vertigo; 2. Slight nausea and a peculiar constrictive feeling at the back of the throat; 3. An unwillingness and almost inability to energise in any way, and sometimes a desire to recline. These feelings were experienced in a few minutes after the dose was taken.

The result of administration in the forty cases in which I have noted the effect, may be represented as follows:

1. Slight, or well marked temporary amelioration, without any decided effect on the cause of the disease. This result I have observed in ten cases: one of puerperal mania, in which the dose was probably insufficient; one of melancholia, in which the treatment was altered; one of menstrual mania; three of acute mania of long standing and great severity, ending in exhaustion and death, and resisting every mode and plan of treatment; two in recent mania, the effect being sustained and cure completed by other means; one in acute mania, when its administration was not sustained; and one in an acute paroxysm of chronic mania.

Even in these cases the effect has been most beneficial, the patient becoming very much more manageable, giving over violence, noise, excitement, stripping, restlessness, etc., and becoming more amenable to moral and dietetic management.

2. A more decided and permanent effect, the disease being still stationary or progressive. Of this class I have noted nineteen; five general paralytics, in whom, while the morbid excitement has been vastly abated or expelled, the disease has progressed to its fatal termination; five chronic maniacs, in whom an inter-current acute paroxysm was effectually dispelled; three melancholics, in whom acute manifestations were permanently removed; one case of acute dementia, in which excessive hypernoia was immediately arrested; two epileptics, in whom a paroxysm of excitement was summarily dismissed; two cases of epilepsy with menstrual excitement, in which the contrast of duration with former attack was most striking; one case of hysterical mania, in which the disease oscillated from an extreme of hypernoia to an extreme of hyponoia; one case of puerperal mania, in which rest and sleep were induced after other measures had signally failed; one case of mania with hemiplegia, in which an intercurrent excitement was disposed of; and one case of mania with chronic hydrocephalus, where a change in conduct and demeanor was very evident.

In all the cases the benefit conferred has been simply obtained, satisfactorily established, and duly appreciated by the attendants and patient.

3. Cases in which the drug has been a factor, and a very main one, in rapid restoration to reason. The cases of this class have been eight in number: six of acute mania, and two of acute melancholia. I shall append some of the most interesting cases of each class.

III. The preparation which I have employed in every case has been Scheele's dilute acid, which I have found remarkably uniform and convenient.

The dose has varied from 5 to 6 minims. Beyond that, disagreeable effects are apt to occur; 5 minims is the most convenient dose, and if the effect is not promptly established, a repetition every quarter of an hour effectually secures it. The effect is rather evanescent, and has been observed in some cases to disappear within an hour; but if a slight degree of hypernoia recurs, a subsequent administration is apt to have a more potent effect, in consequence of a prior. The interval may vary according to the nature and exigencies of the case, and the effect produced. Short at first, until an effect is produced (5 to 15 minutes), it may be prolonged after the excitement has disappeared (to one or two hours). It may, in many cases, be left, within limits, to the discretion of an intelligent attendant.

The only modes of administration I have employed have been mixture and subcutaneous injection. The simplest and best menstruum is water, and 5 minims may be easily and safely introduced beneath the skin, combined with 30 minims of water, by means of Wood's syringe, when the patient resists all other means. Of its application to the extensive pulmonary mucous membrane, by means of pulverization and inhalation, I have no experience; but I should anticipate interesting and important results from shch a method of administering it, and other medicines, in insanity.

IV. The advantages of the drug, in comparison with other calmatives and hypnotics, are:—1. The rapidity, certainty, and simplicity of its effects. 2. Its manageability and freedom from any cumulative property. 3. The absence of any disagreeable, concomitant, or consequent physical disturbance, which most other analogous modes of remedial treatment possess. 4. Its small bulk, want of color, and miscibility. 5. Its want of repulsive smell and taste—a very great virtue with the insane, who are very apt to rebel against medicines. 6. Its not impairing appetite and digestion, but rather improving both.

On the whole, I should recommend and urge the adoption of the drug in every case of insanity with hypernœia, as an empirical antagonist to that pathological phenomenon, combining or exhibiting it simultaneously with any other remedy or plan of treatment which an ascertained pathological condition may demand. Simply as a "quietener" it has its merits, proving an invaluable auxiliary to the moral management of a ward generally, or the patient in particular. Very often, I have heard the attendants express their sense of the great value of "the medicine" as completely altering the character of their gallery and the conduct of their patients—benefiting the latter, and assisting themselves in the performance of their duties. But, in acute cases of mania and melancholia, and in maniacal and melancholic paroxysms, I attach a much higher value to it, and should more strongly advise its trial, as, from the experience I have had, I feel convinced of its potency and efficacy. I have no doubt that it has the power promptly of staying cases running on to chronic insanity on the one hand, or exhaustion and death on the other, and of obtaining, simply and satisfactorily, results which are at present aimed and arrived at by boiling a patient in hot baths, half drowning him in douches, narcotizing with opium or morphia, nauseating with tartar emetic, exhausting with purging, roasting with blister, or debilitating with lancet, leech, or cup.—*Medical Times and Gazette*, 1863.

